# Outcomes of laparoscopic and endoscopic cooperative surgery for gastric submucosal tumors: A retrospective multicenter study at 21 Japanese institutions

**DOI:** 10.1002/ags3.12787

**Published:** 2024-03-13

**Authors:** Yoshikazu Hashimoto, Nobutsugu Abe, Souya Nunobe, Hirofumi Kawakubo, Tetsuya Sumiyoshi, Naohiro Yoshida, Yoshinori Morita, Masanori Terashima, Zenichiro Saze, Manabu Onimaru, Eigo Otsuji, Shu Hoteya, Haruhiro Yamashita, Takashi Fujimura, Tsuneo Oyama, Ken Ohata, Satoki Shichijo, Kazuaki Tanabe, Kiyohiko Shuto, Takashi Ikeya, Hisashi Shinohara, Satoshi Tanabe, Naoki Hiki

**Affiliations:** ^1^ Department of Gastroenterological and General Surgery Kyorin University Faculty of Medicine Tokyo Japan; ^2^ Department of Gastroenterological Surgery Cancer Institute Ariake Hospital Tokyo Japan; ^3^ Department of Surgery Keio University School of Medicine Tokyo Japan; ^4^ Department of Gastroenterology Tonan Hospital Sapporo Hokkaido Japan; ^5^ Department of Gastroenterology Ishikawa Prefectural Central Hospital Kanazawa Japan; ^6^ Department of Gastroenterology Kobe University International Clinical Cancer Research Center Hyogo Japan; ^7^ Division of Gastric Surgery Shizuoka Cancer Center Shizuoka Japan; ^8^ Department of Gastrointestinal Tract Surgery Fukushima Medical University Fukushima Japan; ^9^ Digestive Diseases Center Showa University Koto Toyosu Hospital Tokyo Japan; ^10^ Division of Digestive Surgery, Department of Surgery Kyoto Prefectural University of Medicine Kyoto Japan; ^11^ Department of Gastroenterology Toranomon Hospital Tokyo Japan; ^12^ Department of Gastrointestinal Oncology National Hospital Organization Okayama Medical Center Okayama Japan; ^13^ Department of Surgery Toyama City Hospital Toyama Japan; ^14^ Department of Endoscopy Saku Central Hospital Advanced Care Center Nagano Japan; ^15^ Department of Gastrointestinal Endoscopy NTT Medical Center Tokyo Tokyo Japan; ^16^ Department of Gastrointestinal Oncology Osaka International Cancer Institute Osaka Japan; ^17^ Department of Perioperative and Critical Care Management, Graduate School of Biomedical and Health Sciences Hiroshima University Hiroshima Japan; ^18^ Department of Surgery Teikyo University Chiba Medical Center Chiba Japan; ^19^ Department of Gastroenterology St. Luke's International Hospital Tokyo Japan; ^20^ Department of Gastroenterological Surgery Hyogo Medical University Hyogo Japan; ^21^ Department of Advanced Medicine Research and Development Center for New Medical Frontiers Kitasato University School of Medicine Kanagawa Japan; ^22^ Department of Upper Gastrointestinal Surgery Kitasato University School of Medicine Kanagawa Japan

**Keywords:** gastric deformity, gastric submucosal tumor, gastrointestinal stromal tumor, laparoscopic endoscopic cooperative surgery

## Abstract

**Aim:**

We conducted a multicenter study on classical laparoscopic and endoscopic cooperative surgery (LECS) and LECS‐related procedures to retrospectively clarify the safety, problems, and mid‐term outcomes of these methods after their coverage by the national health insurance.

**Methods:**

A total of 201 patients who underwent classical LECS/LECS‐related procedures for gastric submucosal tumors (G‐SMTs) in 21 institutions affiliated with the Laparoscopy Endoscopy Cooperative Surgery Study Group from April 2014 to March 2016 were included. Data was retrospectively obtained from the patients' charts.

**Results:**

The most common surgical procedure was classical LECS (155 patients, 77.1%), non‐exposed endoscopic wall inversion surgery (22 patients, 11.4%), a combination of laparoscopic and endoscopic approaches to neoplasia with non‐exposure technique (16 patients, 8%), and closed LECS (two patients, 1%). Only six (3%) patients underwent LECS with gastrostomy. The mean operative time and blood loss were 188.4 (70–462) minutes and 23.3 (0–793) g, respectively. Ten (5%) patients developed postoperative complications (Clavien–Dindo classification grade II or higher). Two patients needed reoperation due to postoperative bleeding or anastomotic leakage. All tumors were resected with negative margins. A total of 127 (63.2%) patients underwent follow‐up observations for over 36 months, one of whom had a recurrence of peritoneal dissemination and one had poor oral intake.

**Conclusion:**

Classical LECS and LECS‐related procedures for G‐SMTs have favorable short/mid‐term outcomes.

## Abstract 2

We conducted a multicenter study on classical laparoscopic and endoscopic cooperative surgery (LECS) and LECS‐related procedures to retrospectively clarify the safety, problems, and mid‐term outcomes of these methods after their coverage by the national health insurance. A total of 201 patients who underwent classical LECS/LECS‐related procedures for gastric submucosal tumors (G‐SMTs) in 21 institutions affiliated with the Laparoscopy Endoscopy Cooperative Surgery Study Group from April 2014 to March 2016 were included. Classical LECS and LECS‐related procedures for G‐SMTs have favorable short/mid‐term outcomes.

## 1 INTRODUCTION

Laparoscopic surgery using a linear stapler is a standard surgical procedure for the treatment of gastric submucosal tumors (G‐SMTs); however, whether complete oncologic resection can be achieved with this method remains unclear.^1^, ^2^ G‐SMTs are covered by the normal gastric wall, and the ideal resection line is difficult to determine through laparoscopic observation alone.^1^, ^3^ The linear stapler inevitably excises the round tumor linearly. Therefore, this type of surgery can result in a larger resection than expected and cause stenosis or deformity of the stomach^4^, ^5^ or, conversely, result in a positive surgical margin and cause tumor recurrence.^6^, ^7^

In 2008, Hiki et al.^4^ reported the successful use of an endoscopic submucosal dissection (ESD) technique to assist in laparoscopic local resection of the stomach. This technique, named laparoscopic and endoscopic cooperative surgery (LECS), is a method in which tumor observations are made laparoscopically and endoscopically in order to determine the overall appearance of the tumor. The resection range is determined by performing incisions around the tumor with endoscopic procedures, followed by minimal laparoscopic‐guided resection. The gastric wall defect is sutured laparoscopically in order to prevent gastric deformity. Using this technique, the target area can be resected as minimally as possible. LECS was developed owing to the rarity of lymph node metastasis from gastrointestinal stromal tumors (GISTs), which are the most frequent type of G‐SMTs; hence, local and extensive lymphadenectomy is not required,^2^, ^8^ and the standard treatment is surgical resection with negative margins that do not damage the pseudocapsule of the tumor.^8^, ^9^, ^10^ LECS for the removal of G‐SMTs has been covered by the Japanese national health insurance since 2014 and, since then, has been widely practiced throughout Japan.

Meanwhile, the classical (original) LECS involves opening the gastric wall, which allows the gastric juice to flow into the abdominal cavity, raising concerns about the possibility of intra‐abdominal infections and/or peritoneal dissemination of ulcerated tumors.^3^, ^4^, ^5^, ^11^ Thus, various forms of LECS‐related procedures (also known as non‐exposing LECS) have been developed to address the above issue: non‐exposed endoscopic wall‐inversion surgery (NEWS),^12^, ^13^ a combination of laparoscopic and endoscopic approaches to neoplasia with a non‐exposure technique (CLEAN‐NET),^14^, ^15^ and closed LECS.^16^ These LECS‐related procedures do not involve opening the gastric wall, thus avoiding the spread of gastric contents into the abdominal cavity. A questionnaire survey conducted by the Japan Society for Endoscopic Surgery^17^ showed that classical LECS and LECS‐related procedures have been introduced in many facilities, and their application rate is gradually increasing, particularly after coverage by the national health insurance. However, to the best of our knowledge, no national multicenter research has examined the outcomes of these surgeries after coverage by the National Health Insurance. We considered the necessity of a multicenter study since the outcomes of LECS/LECS variation surgery were expected to vary depending on each facility.

Therefore, we conducted a retrospective multicenter study at 21 Japanese institutions to elucidate the short‐ and mid‐term outcomes of classical LECS and LECS‐related procedures for G‐SMTs after coverage by the Japanese national health insurance.

## 2 METHODS

### 2.1 Patients

A total of 201 patients who underwent classical LECS or LECS‐related procedures for G‐SMTs at 21 institutions affiliated with the Laparoscopy Endoscopy Cooperative Surgery Group from April 2014 to March 2016 were eligible for the study. The Laparoscopy Endoscopy Cooperative Surgery Group was established in 2010. Since its establishment, it has been active as a group of surgeons/endoscopists interested in LECS or LECS‐related procedures. Nine university hospitals, three cancer center hospitals, and nine general hospitals participated in this multicenter study. The number of cases registered at each facility varies from one to 41. The research was approved by the Faculty of Medicine Research Ethics Committee, Kyorin University (H30‐094), and conducted after ethical review at each facility and with informed consent from the patients. The informed consent process followed an opt‐out approach, where participants were provided with detailed information about the study and their participation was presumed unless they explicitly chose to opt‐out. A retrospective case‐series study was then conducted based on the information obtained from the patients' medical records. Data on the patient's background, tumor characteristics, and treatment outcomes were collected.

Patient background data included sex, age, body mass index (BMI), history of upper abdominal surgery and surgical procedures, co‐morbidities, and symptoms. Tumor characteristics included location, growth type, diameter, presence or absence of ulceration, and histopathological findings. The short‐term (operative) outcomes included operative time, estimated blood loss, necessity of small laparotomy incisions of less than 5 cm, presence or absence of conversion to open surgery and its reason, use of the gastric wall defect closure method, time to start oral intake, length of postoperative stay, and postoperative complications (according to the Clavien–Dindo (C‐D) classification^18^). The mid‐term outcomes included the presence or absence of late adverse events and recurrence (type of recurrence and time from surgery to recurrence).

The severity of postoperative complications was graded using the C‐D classification. Because C‐D grade I is regarded as clinically non‐significant, only C‐D grade II or higher (≥C‐D II) were defined as complications in the present study.

Late adverse events included postoperative morbidity after the discharge date. The following events were defined as late adverse events: anastomotic stenosis, dumping syndrome, and poor oral intake.

### 2.2 Data collection and statistical analysis

Data on the patient's background, tumor characteristics, and treatment outcomes were collected and analyzed. All continuous variables except the observation period were expressed as means, standard deviations (SDs), and ranges. All statistical analyses were conducted using SPSS Statistics version 27 for Windows (IBM Corp., Armonk, NY).

### 2.3 Indications for classical LECS and LECS‐related procedures

Classical LECS and LECS‐related procedures are indicated for G‐SMTs of less than 5 cm with or without mucosal ulceration, according to guidelines.^19^ For G‐SMTs, the tumor diameter was preoperatively measured using endoscopy (including endoscopic ultrasonography) and/or CT scans.

### 2.4 Surgical techniques

#### 2.4.1 Classical LECS

Classical LECS was performed as described previously by Hiki et al.^4^ It involves the observation of the tumor location with a laparoscope, the insertion of an endoscope, and the setting of a resection range by poking forceps inside and outside the gastric wall. Before the endoscopic procedures, the attachment of the lesser or greater omentum to the stomach in the excision area was dissected using a laparoscopic electrocautery device. Clamp forceps are applied to the upper jejunum within 5–10 cm of the Treitz' ligament prior to endoscopic manipulation. After severing the blood vessels around the tumor, a circumferential incision as deep as the submucosal layer around the tumor is made from the gastric lumen using the ESD technique using an endoscopic electrocautery device. Subsequently, a full‐thickness incision is performed along the incision line around the tumor using an endoscopic electrocautery device. This is followed by a laparoscopic full‐thickness incision around the remaining circumference, followed by a hand‐sewn closure, closure using a laparoscopic stapling device, or tumor resection with closure of the gastric wall using a laparoscopic stapling device.^4^, ^11^, ^20^, ^21^, ^22^ In this study, the LECS with crown method was also included in the classical LECS. In the LECS with crown method presented by Nunobe et al.,^23^ the tumor is inverted toward the stomach by pulling the suture at the edge of the resection specimen to prevent contact between the tumor and visceral tissue. In this technique, the gastric resection line is pulled up in a bowl shape by several stitches, which looks like a crown.^24^ This technique theoretically minimizes the risk of gastric contents spilling out into the abdominal cavity and prevents the tumor touching any intra‐abdominal tissue.^23^, ^24^

#### 2.4.2 NEWS

NEWS was performed as described previously by Goto et al.^12^ It consists of four major procedures: (1) marking the tumor circumferentially on the mucosal and serosal surfaces using an endoscope and laparoscope, respectively; (2) submucosal injection of sodium hyaluronate with indigo carmine dye using an endoscope; (3) laparoscopic circumferential seromuscular incision with suture closure under laparoscopy; and (4) circumferential muco‐submucosal incision under endoscopy. The resected specimen is then retrieved orally.^12^, ^13^

#### 2.4.3 CLEAN‐NET

Inoue et al.^14^ developed a method of non‐exposed full‐thickness resection after seromuscular incision, preserving the mucosa, which acts as a barrier. In this procedure, after the lesion is marked endoscopically, the mucosal layer is fixed to the seromuscular layer with four full‐layer stay sutures. After submucosal injection of indocyanine green solution, the seromuscular layer is dissected along the outside of the four stay sutures using a laparoscopic electrocautery device. The full‐layer specimen is then lifted by the four stay sutures, and the mucosa surrounding the full‐layer specimen is also pulled up. The full‐layer specimen is dissected with sufficient margins using a laparoscopic stapling device.^14^, ^15^

#### 2.4.4 Closed LECS

Kikuchi et al.^16^ developed a new non‐exposed full‐thickness resection called “closed LECS.” In this procedure, mucosal markings are first made around the tumor, and the marked circumferential area is resected endoscopically. Subsequently, serosal markings are made corresponding to the submucosal dissection line using endoscopic light. Next, a spongy spacer is placed at the center of the suture line on the serosal surface, and a seromuscular suture is made with inversion of the marked lesion and spacer into the inside of the stomach. Finally, circumferential seromuscular dissection is performed.^16^

#### 2.4.5 LECS with gastrostomy

After confirming the submucosal tumor laparoscopically and, if necessary, using gauze to prevent thermal damage to surrounding organs such as the pancreas, a mucosal incision around the tumor is performed endoscopically. Subsequently, a gastrostomy is constructed, a single port is placed, and a full‐thickness incision of the gastric wall is performed endoscopically. In this surgical method, the tumor is resected laparoscopically from the port of the gastrostomy, and the defect is sutured closed.^25^ The surgery with gastrostomy was included as LECS‐related procedure in this study because it consists of endoscopic and laparoscopic procedures.

## 3 RESULTS

A total of 201 patients were recruited, of whom 127 underwent follow‐up observations for at least 3 years. Table 1 shows patient background characteristics. The mean patient age was 60 years (SD: 13.93), with 92 men and 109 women. The mean BMI was 22.8 (SD: 3.23), and 171 patients (85.1%) were asymptomatic. A total of 58 patients (28.9%) had a history of abdominal surgery, while 71 patients (35.3%) had comorbidities. GIST was the most common preoperative diagnosis (167 patients, 83.1%).

**TABLE 1:** Clinical characteristics of patients who underwent classical LECS and LECS‐related procedures for G‐SMTs.

	*N* = 201
Age, years, mean ± SD (range)	60.2 ± 13.9 (18–88)
Sex, M/F	92/109
BMI, kg/m^2^, mean ± SD (range)	22.8 ± 3.23 (15.0–31.8)
Previous abdominal operation	58 (28.9%)
Upper abdominal	6 (3.0%)
Lower abdominal	53 (26.4%)
Upper and lower abdominal	2 (1.0%)
Unknown	1 (0.5%)
Co‐morbidities	71 (35.3%)
Hypertension	54 (26.9%)
Diabetes	23 (11.4%)
Heart disease	13 (6.5%)
Asthma	8 (4.0%)
Symptom	
No symptom	171 (85.1%)
Epigastralgia	14 (7.0%)
Epigastric discomfort	3 (1.5%)
Melena	8 (4.0%)
Hematemesis	1 (0.5%)
Anorexia	1 (0.5%)
Lower abdominal pain	1 (0.5%)
Unknown	2 (1.0%)
Preoperative diagnosis	
GIST	167 (83.1%)
Leiomyoma	6 (3.0%)
Neuroendocrine tumor	2 (1.0%)
Gastric cancer	1 (0.5%)
Others^a^	25 (12.4%)

Abbreviations: BMI, body mass index; GIST, gastrointestinal stromal tumor; G‐SMTs, gastric submucosal tumors; LECS, laparoscopic and endoscopic cooperative surgery; SD, standard deviation.

^a^ Others include ectopic pancreas, monophasic synovial sarcoma, or adenocarcinoma recurrence after endoscopic resection.

Table 2 shows patient surgical outcomes. The mean operative time and estimated blood loss were 188.4 min (range: 70–462 min) and 23.3 g (range: 0–793 g), respectively. No differences were found in operative time, blood loss, or postoperative complication rates based on the number of cases between facilities (data not shown). The operative time tended to be shorter for CLEAN‐NET than for classical LECS and other LECS‐related procedures (mean operation time, 143.2 vs. 192.4 min). The operative time tended to be longer for surgery in the esophagogastric junction/upper third of the stomach than in the middle/lower third of the stomach (mean operation time, 195.0 vs. 177.4 min). Additionally, the operative time tended to be longer for surgery of the tumor located on the posterior wall than for tumors located on others (mean operation time, 211.1 vs. 180.8 min). Classical LECS was the most commonly performed surgical procedure (155 patients, 77.1%), followed by NEWS (22 patients, 11.4%), CLEAN‐NET (16 patients, 8%), and closed LECS (two patients, 1%); meanwhile, LECS with gastrostomy was performed in six patients (3%).

**TABLE 2:** Surgical outcomes of patients (*n* = 201) who underwent classical LECS and LECS‐related procedures for G‐SMTs.

	*N* = 201
Operative time, min, mean ± SD (range)	188.4 ± 67.71 (70–462)
Estimated blood loss, g, mean ± SD (range)	23.3 ± 83.54 (0–793)
Operation type	
Classical LECS	155 (77.1%)
NEWS	22 (11.4%)
CLEAN‐NET	16 (8.0%)
Closed LECS	2 (1.0%)
Intragastric surgery	6 (3.0%)
Assistance for small incisions less than 5 cm	
No	177 (88.1%)
Yes	10 (5.0%)
Unknown	14 (7.0%)
Conversion to open surgery	1 (1.0%)
Postoperative complication	10 (5.0%)
Bleeding	3 (1.5%)
Bloodstream infection	2 (1.0%)
Anastomotic leakage	1 (0.5%)
Delayed gastric emptying	1 (0.5%)
SSI	1 (0.5%)
Hypertension	1 (0.5%)
Urination disorders	1 (0.5%)
Closure of gastric wall defect	
Hand sewing	130 (64.7%)
Stapled	71 (35.3%)
Time until oral intake, days, mean ± SD (range)	3.2 ± 1.74 (1–19)
Length of hospital stay, days, mean ± SD (range)	8.6 ± 4.16 (4–51)

Abbreviations: Classical LECS, classical laparoscopy endoscopy cooperative surgery; CLEAN‐NET, a combination of laparoscopic and endoscopic approaches to neoplasia with a non‐exposure technique; Closed LECS, closed laparoscopy endoscopy cooperative surgery; G‐SMTs, gastric submucosal tumors; NEWS, non‐exposed endoscopic wall‐inversion surgery; SD, standard deviation; SSI, surgical site infection.

Closure of gastric wall defects was performed in 131 patients using hand‐sewn sutures (running sutures: 105; interrupted sutures: 23; running + interrupted sutures: 3). A total of 71 patients (35.3%) underwent closure of gastric wall defects using laparoscopic stapling devices (median number of stapling devices used: 2 [range: 1–5]).

A total of 178 patients (88.1%) did not require imaging assistance when small incisions of less than 5 cm were made. On the other hand, 10 patients (5.0%) required small laparotomy assistance due to the difficulty in suturing gastric wall defects and tumors with mucosal ulcerations. Only one patient (0.5%) required conversion to open surgery due to the difficulty in securing the field of view as the tumor in the fornix was depressed in the lateral segment of the liver.

Ten patients (5%) developed postoperative complications: three had postoperative bleeding, two had bloodstream infections, one had an anastomotic leakage, one had delayed gastric emptying, one had a surgical site infection, one had hypertension, and one had urination disorder. Of the three patients who experienced postoperative bleeding, one had spontaneous hemostasis with blood transfusion alone (C‐D grade II), one needed endoscopic hemostasis (C‐D grade IIIa), and one underwent re‐operative laparoscopic hemostasis (C‐D grade IIIb) to reduce the need for blood transfusion. Moreover, one patient underwent re‐operation (C‐D grade IIIb) due to the occurrence of an intra‐abdominal abscess associated with anastomotic leakage. The other postoperative complications were classified as C‐D grade II. One patient developed delayed gastric emptying (C‐D grade II) during the perioperative period. In this case, classical LECS was conducted for a leiomyoma measuring 25 mm in the greater curvature of the upper‐third of the stomach. Conservative treatment by insertion of a gastric tube enabled oral intake, and the patient was discharged from the hospital on the 21st postoperative day. The mean time to postoperative oral intake was 3.2 days (range: 1–19 days), and the mean postoperative hospital stay was 8.6 days (range 4–51 days).

Table 3 shows the clinicopathological characteristics of tumors. The mean tumor diameter was 30.7 mm (range: 5–80 mm). Regarding tumor maldistribution, patients most commonly had an intraluminal growing type (135, 67.2%). Moreover, 26 patients (12.9%) had mucosal ulceration. The histopathology of the tumors showed the following conditions: GIST, 144 patients (71.6%); leiomyoma, 27 patients; schwannoma, 13 patients; heterotopic pancreas, five patients; neuroendocrine tumor, two patients; gastric cancer, one patient; and others, nine patients (lipoma, gastric adenomyoma, monophasic synovial sarcoma, bronchogenic cyst, granulomatous inflammation (due to Anisakis), fibrous nodule, ectopic gland, and fibrous lesion). All surgical margins were histopathologically negative.

**TABLE 3:** Clinicopathological characteristics of the tumors.

	*N* = 201
Tumor size, mm, mean ± SD (range)	30.7 ± 12.54 (5–80)
Tumor location	
Esophagogastric junction	3 (1.5%)
Upper third of the stomach	123 (61.2%)
Middle third of the stomach	53 (26.4%)
Lower third of the stomach	22 (10.9%)
Tumor location	
Greater curvature	59 (29.4%)
Posterior wall	51 (25.4%)
Anterior wall	47 (23.4%)
Lesser curvature	44 (21.9%)
Growth pattern	
Intraluminal	135 (67.2%)
Intramural	49 (24.4%)
Extraluminal	17 (8.5%)
Mucosal ulceration	
Yes	26 (12.9%)
No	175 (87.1%)
Histopathological margin	
Negative	201 (100%)
Positive	0 (0%)
Pathological diagnosis	
GIST	144 (71.6%)
Very low risk	25 (12.4%)
Low risk	85 (42.3%)
Intermediate risk	19 (9.5%)
High risk	15 (7.5%)
Leiomyoma	27 (13.4%)
Schwannoma	13 (6.5%)
Heterotopic pancreas	5 (2.5%)
Neuroendocrine tumor	2 (1.0%)
Gastric cancer	1 (0.5%)
Others^a^	9 (4.5)

Abbreviations: GIST, gastrointestinal stromal tumor; SD, standard deviation.

^a^ Others include lipoma, gastric adenomyoma, monophasic synovial sarcoma, bronchogenic cyst, granulomatous inflammation (due to Anisakis), fibrous nodule, ectopic gland, and fibrous lesion.

Table 4 shows the mid‐term outcomes of patients who underwent a postoperative follow‐up period of more than 36 months. The median postoperative follow‐up period was 51 months (range: 36–91 months). In all patients, including patients whose follow‐up periods were less than 36 months, regardless of malignant or benign tumor, no recurrence, metastasis, or late adverse events occurred. No patient had anastomotic stenosis or dumping syndrome as a late adverse event from discharge to the third year and over 3 years of observation; however, one patient had a poor oral intake with weight loss. This patient developed a leiomyoma of 58 mm in the lesser curvature of the upper‐third of the stomach but did not develop complications during the postoperative period. The patient was eventually discharged from the hospital on postoperative day 10. Weight loss was 10 kg or 17% of the preoperative weight at 3 months postoperatively, and the greatest loss occurred at 14 months postoperatively, at 14 kg or 22% of the preoperative weight, with gradual recovery subsequently. An upper gastrointestinal endoscopy conducted 5 months postoperatively showed no food residue in the stomach, and an upper gastrointestinal series conducted 9 months postoperatively showed neither gastric deformity nor food residue. Why the patient in this study had poor oral intake remains unclear; however, the involvement of mental factors cannot be denied due to the presence of psychiatric disorders; therefore, the patient's symptoms may not have been only gastric‐related.

**TABLE 4:** Mid‐term outcome of patients after more than 3 years of follow‐up.

	*N* = 127
Late adverse events	1 (0.8%)
Anastomotic stenosis	0 (0%)
Dumping	0 (0%)
Poor oral intake	1 (0.8%)
Recurrence	
No	126 (99.2%)
Yes	1 (0.8%)^a^
Postoperative follow‐up period, months, median (range)	51 (36–91)

^a^ Peritoneal metastasis.

Disseminated recurrence was observed in one patient at 18 months post‐surgery (classical LECS), and the patient died of the original disease at 86 months post‐surgery. This patient, who had an intramural tumor with a maximum diameter of 52 mm in the posterior wall of the upper gastric body, underwent classical LECS. Histopathological examination of the resected specimen confirmed the diagnosis of GIST, and the mitotic image was 31/50 HPF (high power field), indicating a high risk according to the Fletcher classification.^26^ Regarding the type of recurrence, tumor formation in the pelvic cavity occurred at 18 months post‐LECS, which was consistent with the dissemination pattern of GIST.

## 4 DISCUSSION

We collected 201 patients who underwent classical LECS and LECS‐related procedures for G‐SMTs in 21 facilities across Japan when national health insurance coverage was available. We then conducted a retrospective review of the surgical and mid‐term outcomes.

Laparoscopic local resection of the stomach for gastric GIST, which was conducted before the development of LECS, has a recurrence rate of 4%–5%.^6^, ^7^ Our study reported a recurrence rate of 0.8%, with only one patient in 127 patients who were followed up for more than 3 years. In the present patient, recurrence was probably not caused by the procedure (classical LECS) but due to the patient having a high‐risk GIST tumor with an increased risk of recurrence. Moreover, histopathological examination revealed that all the tumors were resected with negative margins. Therefore, the present study demonstrated that classical LECS and LECS‐related procedures may be oncologically safe when compared with conventional laparoscopic local resections of the stomach.

Regarding operative time, estimated blood loss, and postoperative complication frequency, the results were almost similar to those reported in other studies.^3^, ^4^, ^14^, ^15^, ^16^, ^20^, ^21^, ^22^, ^27^ However, the relatively longer operative time for LECS than for laparoscopic partial gastrectomy without lymphadenectomy may be caused by the addition of endoscopic procedures.

A detailed examination of 10 patients (5%) who developed postoperative complications of C‐D grade II or higher included two of the three patients who experienced bleeding and underwent either endoscopic (C‐D grade IIIa) or laparoscopic hemostasis (C‐D grade IIIb), all three patients underwent classical LECS, and the gastric wall defect was closed by hand‐sewn stitches. In another case, spontaneous hemostasis (C‐D grade II) was achieved through simple blood transfusion, and a laparoscopic stapling device was used to close the gastric wall defect. Additionally, for anastomotic failure (C‐D grade IIIb), hand‐sewn stitching was preferred. Therefore, complications related to the closure of these gastric wall defects may be alleviated by using laparoscopic stapling devices. In April 2022, the use of laparoscopic stapling devices was included in the national health insurance coverage; hence, further improvements in surgical results can be expected in the future.

Infection‐related complications were observed in three patients; two patients underwent classical LECS, while one patient underwent LECS with gastrostomy. No crown technique was performed in these surgeries. Therefore, during these procedures, gastric contents may have spilled into the abdominal cavity and contributed to the occurrence of postoperative inflammation. Therefore, CLEAN‐NET, NEWS, closed LECS, or LECS using the crown method that lifts the gastric wall defect into a bowl‐like shape and prevents the tumor and gastric juice from spilling into the abdominal cavity^23^, ^24^ is expected to reduce the risk of postoperative inflammation and infection‐related complications.

Delayed gastric emptying refers to a disorder caused by the loss of gastric function and occurs in 20% of patients, with vagotomy, pyloric resection, and gastric bypass.^28^ Even after partial gastrectomy, the motility and physiological functions of the remnant stomach on the side of the lesser curvature significantly change compared with those on the side of the greater curvature^29^; the reason for this is that the excision of the Latarjet branch of the vagal nerve, which is distributed in the lesser curvature, impairs remnant gastric motility.^22^ Thus, the reason why the patient in this study developed delayed gastric emptying remains unclear.

Meanwhile, delayed gastric emptying after gastrectomy improves within 10 weeks after surgery.^30^ The perioperative delayed gastric emptying observed in this study occurred within 3 weeks post‐surgery; therefore, a delay in gastric emptying after LECS is unlikely to occur.

A comparison of the multicenter research focusing on classical LECS for G‐SMTs in surgical cases from 2007 to 2011 by Matsuda et al.^27^ and the present study shows that although the incidence of C‐D grade II or higher complications remains unchanged (4.8% and 5%), the complication rate decreased from 1.6% to 0.5% for anastomotic failure and from 1.6% to 0.5% for delayed gastric emptying. Each facility has accumulated experience in performing classical LECS and LECS‐related procedures and has considered minimizing the takedown procedure of the lesser curvature of the omentum. Meanwhile, the present study is the first to examine patients with mid‐term late adverse events associated with these procedures. Although this was a mid‐term observation over 3 years, late adverse events, which are thought to be caused by LECS, are an extremely rare condition.

The present study has some limitations. First, LECS, which needs to be performed by endoscopists and surgeons, cannot be conducted in all facilities nationwide due to the shortage of doctors and the lack of skilled doctors. Second, only 127 of the 201 patients (63.2%) were available for follow‐up observation of at least 36 months. The reason for the restriction is that tracking may not be possible at those facilities because it can only be done at a limited number of facilities. In the future, if the number of facilities where LECS can be performed increases, this restriction will disappear because each facility can follow up. Third, the level of experience differed depending on the facility. Finally, the study was retrospective in nature.

## 5 CONCLUSIONS

Classical LECS and LECS‐related procedures for G‐SMTs have favorable short/mid‐term outcomes; however, postoperative complications, late adverse events, and recurrence may occur in few patients. Preventing gastric juice from spilling into the abdominal cavity, i.e., LECS with crown method, may reduce dissemination recurrence and postoperative inflammatory complications. Further studies will be needed to clarify these merits of LECS with crown method.

## AUTHOR CONTRIBUTIONS

Conception and design of the study: Yoshikazu Hashimoto and Nobutsugu Abe. Acquisition, analysis, and interpretation of data: Yoshikazu Hashimoto, Nobutsugu Abe, Souya Nunobe, Hirofumi Kawakubo, Tetsuya Sumiyoshi, Naohiro Yoshida, Yoshinori Morita, Masanori Terashima, Zenichiro Saze, Manabu Onimaru, Eigo Otsuji, Shu Hoteya, Haruhiro Yamashita, Takashi Fujimura, Tsuneo Oyama, Ken Ohata, Satoki Shichijo, Kazuaki Tanabe, Kiyohiko Shuto, Takashi Ikeya, Hisashi Shinohara, Satoshi Tanabe, and Naoki Hiki. Drafting of the manuscript: Yoshikazu Hashimoto. Critical revision for important intellectual content: Nobutsugu Abe and Naoki Hiki. Final approval of the manuscript: Yoshikazu Hashimoto and Nobutsugu Abe. Acquisition, analysis, and interpretation of data: Yoshikazu Hashimoto, Nobutsugu Abe, Souya Nunobe, Hirofumi Kawakubo, Tetsuya Sumiyoshi, Naohiro Yoshida, Yoshinori Morita, Masanori Terashima, Zenichiro Saze, Manabu Onimaru, Eigo Otsuji, Shu Hoteya, Haruhiro Yamashita, Takashi Fujimura, Tsuneo Oyama, Ken Ohata, Satoki Shichijo, Kazuaki Tanabe, Kiyohiko Shuto, Takashi Ikeya, Hisashi Shinohara, Satoshi Tanabe, and Naoki Hiki.

## FUNDING INFORMATION

Not applicable.

## CONFLICT OF INTEREST STATEMENT

N.H. is Chief Associate Editor of *Annals of Gastroenterological Surgery*. N.H. was supported by grants or donations from Abbott Japan LLC, Chugai Pharmaceutical Co., Ltd., Miyarisan Pharmaceutical Co. Ltd. and Terumo Corporation. The funding source had no role in the design, practice, or analysis of this study. E.O. is Editorial Board Member of *Annals of Gastroenterological Surgery*. T.F. is a reviewer of *Annals of Gastroenterological Surgery*. They will not be involved in the review of this paper or in the selection of reviewers. The other authors declare no conflicts of interest for this article.

## ETHICS STATEMENT

Approval of the research protocol: The protocol for this research project has been approved by a suitably constituted Ethics Committee of the institution, and it conforms to the provisions of the Declaration of Helsinki (Committee of the Faculty of Medicine Research Ethics Committee, Kyorin University, Approval No. H30‐094).

Informed Consent: The study was conducted after ethical review at each facility and with informed consent from the patients.

Registry and the Registration No. of the study/trial: N/A.

Animal Studies: N/A.
